# Bitter friends are not always toxic: The loss of acetic acid bacteria and the absence of *Komagataeibacter* in the gut microbiota of the polyphagous fly *Anastrepha ludens* could inhibit its development in *Psidium guajava* in contrast to *A. striata* and *A. fraterculus* that flourish in this host

**DOI:** 10.3389/fmicb.2022.979817

**Published:** 2022-09-28

**Authors:** Manuel Ochoa-Sánchez, Daniel Cerqueda-García, Andrés Moya, Enrique Ibarra-Laclette, Alma Altúzar-Molina, Damaris Desgarennes, Martín Aluja

**Affiliations:** ^1^Red de Manejo Biorracional de Plagas y Vectores, Clúster Científico y Tecnológico Biomimic^®^, Instituto de Ecología, A.C., Xalapa, Mexico; ^2^Instituto de Biología Integrativa de Sistemas (I2SysBio), Universidad de Valencia and Consejo Superior de Investigaciones Científicas (CSIC), Valencia, Spain; ^3^Fundación para el Fomento de la Investigación Sanitaria y Biomédica de la Comunidad Valenciana (FISABIO), Valencia, Spain; ^4^Red de Estudios Moleculares Avanzados, Clúster Científico y Tecnológico Biomimic^®^, Instituto de Ecología, A.C., Xalapa, Mexico; ^5^Red de Biodiversidad y Sistemática, Clúster Científico y Tecnológico Biomimic^®^, Instituto de Ecología, A.C., Xalapa, Mexico

**Keywords:** *Anastrepha*, *Psidium guajava*, *Acetobacteraceae*, 16S rRNA, gut microbiota, gut dysbiosis, microbe-tephritid interactions

## Abstract

The gut microbiota is key for the homeostasis of many phytophagous insects, but there are few studies comparing its role on host use by stenophagous or polyphagous frugivores. Guava (*Psidium guajava*) is a fruit infested in nature by the tephritids *Anastrepha striata* and *A. fraterculus*. In contrast, the extremely polyphagous *A. ludens* infests guava only under artificial conditions, but unlike *A. striata* and the Mexican *A. fraterculus*, it infests bitter oranges (*Citrus x aurantium*). We used these models to analyze whether the gut microbiota could explain the differences in host use observed in these flies. We compared the gut microbiota of the larvae of the three species when they developed in guava and the microbiota of the fruit pulp larvae fed on. We also compared the gut microbiota of *A. ludens* developing in *C*. *x aurantium* with the pulp microbiota of this widely used host. The three flies modified the composition of the host pulp microbiota (i.e., pulp the larvae fed on). We observed a depletion of Acetic Acid Bacteria (AAB) associated with a deleterious phenotype in *A. ludens* when infesting *P. guajava*. In contrast, the ability of *A. striata* and *A. fraterculus* to infest this fruit is likely associated to a symbiotic interaction with species of the *Komagataeibacter* genus, which are known to degrade a wide spectrum of tannins and polyphenols. The three flies establish genera specific symbiotic associations with AABs. In the case of *A. ludens*, the association is with *Gluconobacter* and *Acetobacter*, but importantly, it cannot be colonized by *Komagataeibacter*, a factor likely inhibiting its development in guava.

## Introduction

The gut of most living organisms harbors complex microbial communities, which are involved in multiple processes of the host’s biology (Wu and Wu, 2012; Cénit et al., 2014; Shin et al., 2015; Chen et al., 2016). In insects, the gut microbiota is frequently associated with essential functions for the host, as occurs in bees (Kwong and Moran, 2016), olive fly (Capuzzo et al., 2005; Ben-Yosef et al., 2010, 2015), flea beetles (Shukla and Beran, 2020) and bark beetles (Adams et al., 2013). In some phytophagous insects, certain components of the gut microbiota are essential for survival, as is the case with the olive fly, *Bactrocera oleae* (Gmelin) (Diptera: Tephritidae) and its obligate endosymbiont “*Candidatus* Erwinia dacicola”. This bacteria is essential for *B. oleae* larvae, playing a key role for its development in the immature olive (Capuzzo et al., 2005; Ben-Yosef et al., 2015), as unripe olives contain high concentrations of secondary metabolites that are lethal to the *B*. *oleae* larva, such as oleuropein (Gutierrez-Rosales et al., 2012). “*Candidatus* Erwinia dacicola” can metabolize oleuropein, allowing the fly larva to feed on unripe olives and successfully complete their development (Shukla and Beran, 2020). Recently, Aluja et al. (2021), while studying the gut microbiota of *Anastrepha ludens* Loew (Diptera: Tephritidae) larvae and adults stemming from six natural hosts including the native ancestral *Casimiroa edulis* La Llave and *C. greggii* (S. Watson) F. Chiang (both Rutaceae), the exotics *Mangifera indica* L. cv. Ataulfo (Anacardiaceae), *Prunus persica* (L.) Batsch (Rosaceae), and *Citrus* x *aurantium* L. (Rutaceae), and the occasional native host *Capsicum pubescens* Ruiz and Pav. cv. Manzano (Solanaceae), observed a decrement in the relative abundance of representatives within the Acetobacteraceae, followed by an increment of representatives within the Enterobacteriaceae in larvae developing in certain hosts. This shift in the gut microbiota, with an increment in the ratio Enterobacteriaceae/Acetobacteraceae was most apparent in the marginal host *C. pubescens*, most likely leading to the poor larval development and high fitness costs observed by Birke and Aluja (2018).

Tephritid flies are a highly diverse group of insects with over 5,000 species described so far (Norrbom et al., 2018). A small proportion of these species are key pests worldwide causing significant economic losses *via* direct damage to the fruit (larvae feeding in them render the fruit unmarketable) or indirectly *via* severe trade restrictions (Aluja and Mangan, 2008). A relevant genus among the Tephritidae group is the genus *Anastrepha*, which is endemic to the Neotropical region (Aluja, 1994), with over 300 species identified so far (Norrbom et al., 2018). In Mexico, and several countries in Central America, *A. ludens* is the most economically important species attacking various citrus species (e.g., *Citrus x sinensis* L. Osbeck, *C*. *x aurantium* L.) and mango (*M. indica*), as well as many other commercial fruit and vegetables (e.g., manzano pepper, *C. pubescens*) (Birke et al., 2015). Importantly for the purposes here, the limit to the extreme polyphagy of *A. ludens* is represented by guava (*Psidium guajava*), a chemically defended fruit widely attacked by *A*. *striata* and the Mexican form of *A*. *fraterculus* (Hernandez-Ortiz, 1993). In the case of *A*. *ludens*, only artificial infestations in fully ripe guavas under field cage or laboratory conditions have been possible, and even under such artificial conditions, the negative effect on larval and adult fitness were highly significant (Birke et al., 2015; Birke and Aluja, 2018). Under these conditions, few larvae survived, their development was slow, and their pupal weight was significantly lower when compared to pupae originating from natural hosts such as *C. edulis* (ancestral host), *C*. *x aurantium* or *M. indica* (Birke et al., 2015; Birke and Aluja, 2018). In contrast, *A. striata* is a stenophagous tephritid species specialized in infesting *Psidium* spp., while the Mexican form of *A*. *fraterculus* is unable to attack citrus (Aluja et al., 2003) [it does so in South America (Salles, 2000)], but infests various species within *Psidium*, *Syzygium jambos* and *Prunus persica* (Aluja et al., 2000; Sivinski et al., 2004). Although the simultaneous occurrence of *A. striata* and *A. fraterculus* in *P*. *guajava* is common (Sivinski et al., 2004), infestation occurs at different phenological stages of the fruit (Birke et al., 2015; De Oliveira et al., 2015). While *A. striata* infests hard, immature guavas that are chemically astringent with high levels of tannins (Lee et al., 2010), *A. fraterculus* prefers stages closer to maturation, with a higher sugar content and lower levels of polyphenols (De Oliveira et al., 2015).

Following the “call for further research on this underappreciated component of insect plant (and enemy) interactions” by Hammer and Bowers (2015), here we compared the gut microbiota of wild *A. striata* and *A. fraterculus* larvae developing in *P. guajava*, plus *A. ludens* larvae artificially developing in this fruit. The gut microbiota of *A. ludens* larvae developing in the natural host *C. x aurantium* was also analyzed to contrast the composition of the gut microbiota in a natural and a conditional/artificial host (*sensu* Aluja and Mangan, 2008). In both cases, we also analyzed the pulp surrounding the larvae. We predicted that the inability of *A*. *ludens* to infest *P*. *guajava* in nature reported by Birke (Birke et al., 2015) could be due, in part, by a dysbiotic larval gut microbiota caused by the deleterious guava pulp rife with polyphenols or the lack of adequate bacterial associations enabling the metabolization of toxic pulp components.

## Materials and methods

### Collection of biological material

Wild *A. striata*, *A. fraterculus* and *A. ludens* larvae were collected from mature fruit with signs of infestation in the orchard “Finca Costa Rica” (Tuzamapan, Veracruz 19°23′56.58″N, 96°53′11.79″W, 834 masl). Additionally, we collected *A*. *ludens* from Teocelo, Veracruz (19°24′57.26″N, 96°58′18.74 ″W, 1,193 masl). Guavas were harvested from various trees and were used to obtain *A. striata* and *A. fraterculus*. Meanwhile, bitter oranges (*C*. *x aurantium*) were harvested to obtain *A. ludens*. In both cases, the fruit were transported to the laboratory and immediately dissected to search for third instar larvae.

Since it is common to find *A. striata* and *A. fraterculus* simultaneously infesting the same guava fruit (Sivinski et al., 2004), all larvae were identified to species. In the third instar, *A. striata* larvae exhibit a clear division in the anal lobe, which is missing in the larvae of *A. fraterculus*, leading to a reliable differentiation of both species (Steck et al., 1990). There was no need to identify the larvae found in bitter oranges as in Mexico only *A. ludens* infests citrus (Aluja et al., 2000). Voucher specimens of each species were kept in INECOL’s Biorational Pest and Vector Management Network (RMBPV).

To characterize the microbiota of fruit where larvae develop (larval niche) and determine the likely microbial exchange between the insect and the fruit, we also collected fruit pulp surrounding the larvae. Both larvae and pulp samples were frozen in liquid nitrogen and stored at −80°C until processing.

### Forced infestation of guavas by *A. ludens*

Forced infestation tests were run in an abandoned guava orchard in Piedra de Agua, Veracruz (19°33′15.54 ″ N, 96°57′46.89 ″ W, 1,531 masl), with *A. ludens* adults originating from infested bitter oranges (*C.* x *aurantium*) collected in neighboring localities. Procedures for fruit handling and harvesting of pupae/adults are described in detail in Aluja et al. (2000). Once the *A*. *ludens* adults emerged, they were kept in 30 × 30 × 30 cm^3^ plexiglass cages, covered with Teflon mosquito netting. They were supplied with water and an artificial diet *ad libitum*. The diet consisted of hydrolyzed protein (MP Biomedicals, Agrisent de Mexico S.A. de C.V., Mexico) and refined cane sugar in a 1:3 ratio. The flies were kept for 15 days at 27 ± 1°C, 63 ± 5% RH and 12:12 h light: dark photoperiod until they reached sexual maturity. Females were not supplied with an oviposition substrate to promote the accumulation of eggs in their ovaries and thus facilitate oviposition into the non-natural host guava (Aluja and Mangan, 2008).

Uninfested guavas were obtained in the field as follows: fruiting trees of a manageable size were identified in the above mentioned abandoned guava orchard and some fruit-bearing branches were covered with organza cloth bags several weeks prior to the bioassay (at this stage fruit were very unripe and not suitable for infestation by either *A*. *striata* or *A*. *fraterculus*). We inspected all fruit to make sure that they did not show signs of infestation by *Conotrachelus* (Coleoptera: Curculionidae: Molytinae), a beetle commonly infesting guavas in the study region (Palemon-Alberto et al., 2021). Two-week-old sexually mature, mated *A*. *ludens* adults, stemming from the oranges previously collected in the field, were transported to the study site in Piedra de Agua from our laboratory at INECOL’s headquarters. Following the protocols of Aluja et al. (2000) and Birke et al. (2015), six sexually mature *A. ludens* specimens in perfect physical condition were selected (i.e., intact wings, with active locomotor activity). These flies were released into the fruit-bearing branches we had previously covered to preclude wild flies and beetles from laying eggs into them. The sex ratio of flies in each bag was 1:1, and the amount of fruit in each bag was between two and four. At the moment of fly release, the guavas were at the ‘yellow in transition’ maturity stage (Birke et al., 2015). Females were allowed to oviposit into guavas for three days and thereafter fruit were monitored daily to detect any that had naturally detached from the branch. Once natural detachment occurred, fruit were collected and transported to the laboratory where they were kept under the conditions described above. Fruit was dissected when they began to show signs of decay. Larvae were immediately processed as described in what follows.

### Sample preparation

Larvae from guavas and oranges were superficially sanitized by the following series of washes: 1-min wash in 500 μl of washing solution (SDS 1%, Tris 10 mM and NaCl 10 mM), followed by a 1-min wash in 500 μl of 1% commercial sodium hypochlorite, and a 1-min wash in 500 μl of 70% ethanol. Finally, two 1-min washes were performed with 500 μl of sterile distilled water. After sanitization, the digestive tracts were dissected from the proventriculus to the terminal region of the hindgut. The dissection of larvae was performed with sterile forceps using a stereoscope (Nikon SMZ 1500, Tokyo, Japan). The sample unit was defined as a pool of five gut tracts. In addition, five mg of the pulp surrounding each larva were collected. Then, pools of 25 mg were obtained for each pulp sample. In turn, each sample unit had five replicates. Samples of larvae guts were kept in 450 μl of RNAlater™ Stabilization Solution (Thermo Scientific^©^) until processing within 24 h of collection.

### gDNA extraction and 16S rRNA gene sequencing

Once the RNAlater™ was removed, all samples were deep frozen in liquid nitrogen and pulverized with a sterile pistil. gDNA extraction was performed with the QIAamp DNA Mini Kit from QIAGEN^©^ (Hilden, Germany). The gDNA obtained was used for the amplification of the 16S rRNA gene. Primers for 16S rRNA gene v3-v4 amplification were selected from Klindworth et al. (2013) and adapted according to the 16S metagenomic sequencing library preparation guide^1^ : 16S Amplicon PCR Forward 5′ TCGTCGGCAGCGTCAGATGTGTA TAAGAGACAGCCTA CGGGNGGCWGCAG and 16S Amplicon PCR Reverse 5′ GTCTCGTGGGCTCGGAGATGTGTATAAGAGACAGGACT ACHVGGGTATCTAATCC. PCR amplification was performed using an enrichment strategy. The enrichment consisted of an initial amplification of 15 cycles, starting from 300 ng of gDNA. Then, 2 μl of PCR1 were used as a template to carry out a PCR2 of 25 cycles. The composition of PCR1 (25 μL) and PCR2 (50 μL) consisted of Qiagen buffer 1X, dNTPs 0.2 mM, MgCl_2_ 0.1 μM, 16s Amp F and R 0.2 μM each one, and Taq Polymerase 0.05 U, with an amplification program of 94 °C/2 min, 15 (PCR1) or 25 (PCR2) cycles of 94 °C/15 seg, 55 °C/30 seg y 72 °C/1 min, and finally 72 °C/5 min. Amplicons were purified with the Promega Wizard^®^ SV Gel and PCR Clean-Up System kit and their concentration was determined by a Qubit 2.0 Fluorometer (Thermo^®^, United States). Purified PCR products were indexed with Ilumina^©^ sequencing adapters using the Nextera XT Index Kit from Illumina^©^ and were purified and quantified as previously described. The quality of the library was determined using the Agilent Bioanalyzer 2100^®^ system. The indexed amplicons were sequenced in a paired-end format (2 × 300 bp) using a MiSeq Reagent Kit V3 (600 cycles), on a MiSeq platform from Illumina^©^. Sequencing was carried out by the Sequencing Unit of the BioMimic™ Scientific and Technological Cluster at the Instituto de Ecologia, A.C.—INECOL.

### Bioinformatic and statistical analyses

The raw reads in paired-end layout (2 × 300) were processed in the QIIME2 (v. 2020.6) platform (Bolyen et al., 2019). We used the dada2 (Callahan et al., 2016) plugin to denoise and resolve the amplicon sequences variants (ASVs) with the following parameters: for forward reads, trimming in the position 20 at the 5′ end and truncating to a length of 270 pb; for reverse reads, trimming in the position 20 at the 5′ end and truncating to a length of 200 pb; removing the chimeric reads with the “consensus” method; the other parameters were used as default. The representative sequences of the resolved ASVs were classified with the classify-consensus-v-search plugin (Rognes et al., 2016), using the SILVA v.132 database as reference (Quast et al., 2013). A phylogeny of the representative sequences of ASVs was built, using the align-to-tree-mafft-fasttree plugin, which uses MAFFT (Katoh, 2002) for the alignment, and FastTree2 to build the phylogeny (Price et al., 2010). All data, the abundance table and phylogeny, were exported to the R environment.

In R (v. 4.1.2), we used the phyloseq (McMurdie and Holmes, 2013) package to perform the diversity analysis. Our sampling protocol allowed for the detection of bacteria inhabiting both, lumen and intracellular gut microbial niches, as we dissected the gut of individuals under aseptic conditions, avoiding pulling other organs/structures. We ended with a clean gut, and therefore are confident that the bacterial DNA studied stems only from gut tissue. So, we sampled the microbiota and endosymbionts. Therefore, first we filtered out the plastid and mitochondrial ASVs. Then, upon the taxonomic classification, we separated the complete data set in two analyzable subunits: gut microbiota and endosymbionts (ASVs classified as within *Wolbachia*) which were analyzed separately. To gain insight into specific species of ASVs classified within *Wolbachia*, we performed a BLASTn search against the SILVA database (v132) with a cutoff of e-value < 0.0003 and “-max_target_seqs” 3. To correct the bias related to the different size in the sample counts, both data sets were normalized with the cumulative sum scaling (CSS) method using the metagenomeSeq package (Paulson et al., 2013). The composition at class and genus level was scaled to relative abundance and visualized with bar plots using the ggplot2 package (Wilkinson, 2011). The alpha diversity indexes, observed species, and Shannon were then calculated. To assess the significant differences in beta diversity between gut microbiota in flies, and between pulp and gut microbiota, the weighted and unweighted UniFrac distance matrixes were calculated, and a Permutational Multivariate Analysis of Variance (PERMANOVA) was applied with the vegan package (Oksanen, 2015) in one way and pairwise mode; *p*-values in pairwise mode were adjusted with the Benjamini–Hochberg (BH) method. A Principal Coordinate Analysis was plotted to visualize the ordination of samples based on UniFrac distance metrics (beta diversity). With the aim of detecting differentially abundant genera in the gut microbiota between species, we also performed a linear discriminant analysis Effect Size (LEfSe) (Segata et al., 2011). Statistical significance was set to a *p*-value < 0.05.

## Results

### General characteristics of the gut microbiota of *A. striata*, *A. fraterculus* and *A*. *ludens*

**FIGURE 1:**
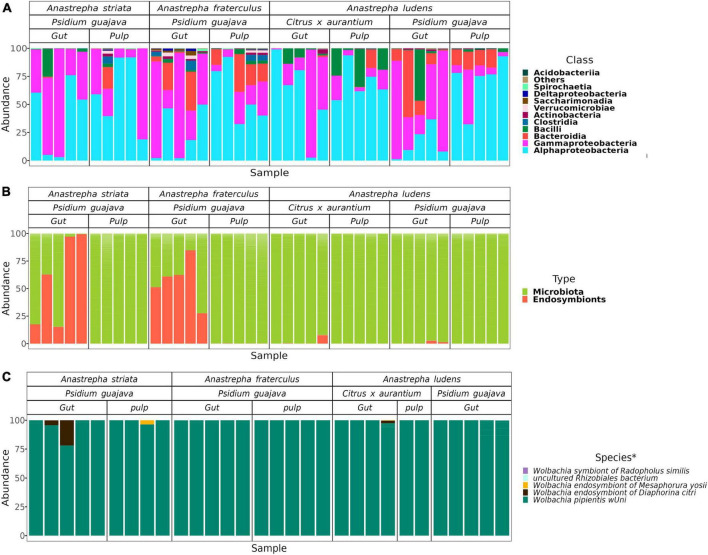
Relative abundance of the microbial components in the gut of *Anastrepha striata*, *A*. *fraterculus* and *A*. *ludens* and the microbiota of pulp where they feed on. **(A)** Relative abundance at the class level of the gut and pulp microbiota; classes with relative abundance lower than 1% were placed in the “Others” category; **(B)** Composition of both types of microbial components in the gut in all samples; **(C)** Relative abundance of the endosymbionts in the samples where they were present. In the pulp of *C. aurantium* there were only a mean of 9 *Wolbachia* reads (0.0156% of the complete data set) and therefore these extremely low numbers do not appear in **(B)**. *The species related to the ASVs of endosymbionts were identified by sequence similarity *via* a BLAST search.

The entire data set comprised 4,091,529 high quality reads, distributed in 40 samples with a mean of 102,288 reads. The data set with only the gut and pulp microbiota (excluding the endosymbiont *Wolbachia*) was composed of 3,398,023 reads, with a sample mean of 84,951 reads. Considering all samples, we detected a total of 1,884 ASVs, distributed among 19 phyla, and 29 classes. Within the wide taxonomic spectrum, 11 classes exhibited more than 1% of relative abundance in at least one sample (Figure 1A). Of the latter, three classes were dominant (i.e., with mean abundances over 1%): Alphaproteobacteria (49.8%), Gammaproteobacteria (34.1%), and Bacilli (5.1%).

The data set with only *Wolbachia* amplicons was composed of 693,473 reads, having very different coverage between the three flies (Figure 1B). In the gut of *A. ludens* larvae that fed on guava pulp, the mean number of reads was 1,181, while in the guava pulp *Wolbachia* was not detected. The gut of *A*. *ludens* larvae that fed on *C*. x *aurantium* pulp, had a sample mean of 1,378, while in the pulp *Wolbachia* was barely detected in very low counts, with a sample mean of 9 reads. Conversely, the guts of *A. striata* and *A. fraterculus*, both feeding on guava pulp, had similar compositions with high *Wolbachia* counts (sample mean of 68,036), but in the pulp, counts were very low, with a sample mean of 58. That is, the few *Wolbachia’s* detected in the pulp of guava most likely stemmed from the feces of the larvae, as opposed to be naturally living there. In this data set, we detected 40 ASVs. The most abundant ASVs where closely related or belonging to *Wolbachia pipientis wUni* (having a 96–99% of sequence similarity, Supplementary Table 1), with a mean relative abundance of 98.9%. The other three species of *Wolbachia*, classified in the SILVA database were: *Wolbachia endosymbiont of Diaphorina citri*, *Wolbachia endosymbiont of Mesaphorura yosii*, and *Wolbachia ensymbiont of Radopholus similis*, all present in low abundances, with a mean of less than 1% (Figure 1C).

### The microbiota of *A. striata*, *A. fraterculus* and *A*. *ludens* developing in *P. guajava*

**FIGURE 2:**
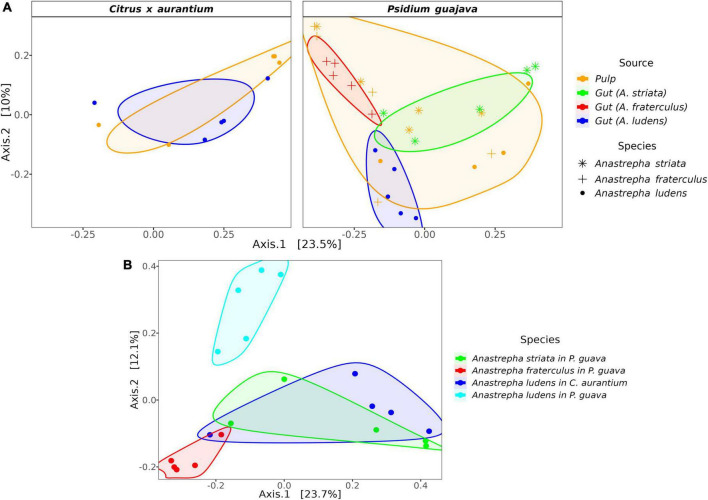
PCoA based on the Unweighted UniFrac distance metric. **(A)** PCoA showing the ordination of the gut and pulp microbiota but separated by host plant; samples of pulp where each species feed on are more like their gut microbiota; **(B)** PCoA only showing the ordination of the gut microbiota and illustrating how *A. ludens* separated from the rest when reared on *P. guajava*. Note that the microbiota of larvae stemming from *C. x aurantium* is more like *A. striata*.

The microbiota composition of the three fly species was significantly different when they fed on *P. guajava*. The PERMANOVA test based on weighted and unweighted UniFrac distances detected 27 and 31% of variance, respectively, explained between species (Supplementary Table 1). However, when the PERMANOVA was run in a pairwise mode (one to one), only the unweighted metric detects significant differences among the three species, explaining a range of variance from 23 to 28% (Table 1). Likewise, the PCoA analysis showed a clear separation in three clusters (Figure 2). By contrast, the pulp microbiota was not significantly different with respect to the gut microbiota in the alpha (Supplementary Figure 1 and Supplementary Table 2) and beta diversity (Figure 2 and Supplementary Table 2).

**TABLE 1:** Pairwise PERMANOVA comparisons of the gut microbiota of *Anastrepha ludens, A. striata*, and *A. fraterculus* larvae stemming from *P. guajava* or *C. x aurantium*.

Gut microbiota in *P. guajava*			
Comparison	F. model	R^2^	adj. *P*-value
*A. striata / A. fraterculus*	2.74	0.25	0.04
*A. striata / A. ludens*	2.43	0.23	0.02
*A. fraterculus / A. ludens*	3.26	0.28	0.02
**Gut microbiota of *A. ludens* in *C. x auriantum***			
**Comparison**	**F. model**	**R^2^**	**adj. *P*-value**

*A. ludens* / *A. striata*	1.09	0.12	1
*A. ludens* / *A. fraterculus*	3.28	0.29	0.04

P-values were adjusted with the BH method.

**FIGURE 3:**
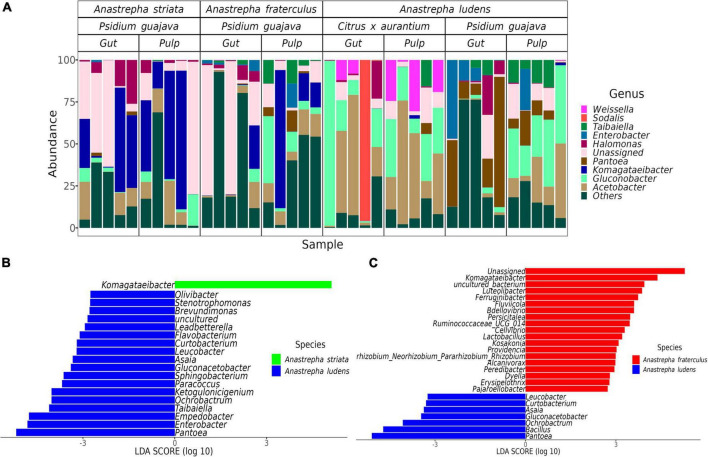
Microbiota composition at genus level and the differences when the flies feed on *P. guajava*. **(A)** Relative abundance of the top 10 genera present in all samples; remaining genera where agglomerated in the “Others” category. Differential genera detected by the LEfSe analysis in paired comparisons of *A. ludens* with *A. fraterculus* **(B)** and *A. striata* **(C)**.

**FIGURE 4:**
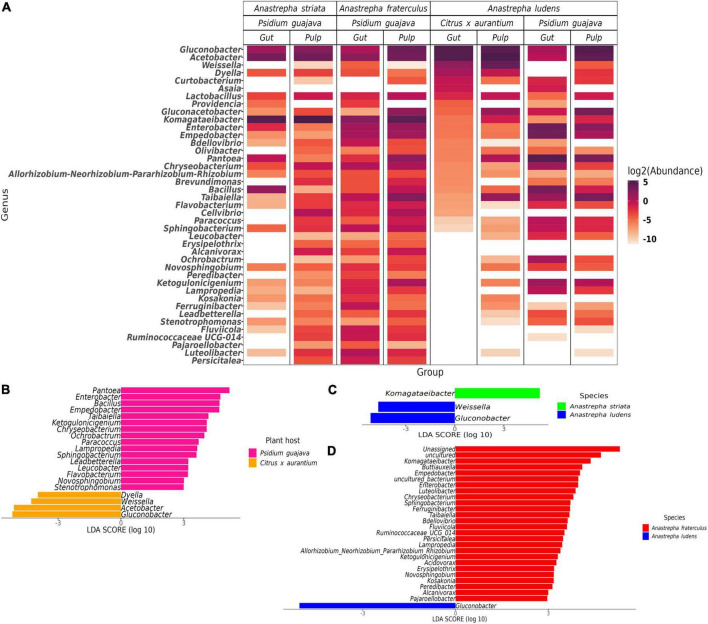
Differences in the normal gut microbiota of *A. ludens* in *Citrus* x *aurantium* compared with *A. striata*, *A. fraterculus* and its dysbiotic state in *P. guajava*. **(A)** Heatmap of all differential genera identified among the three fly species in all samples; **(B)** Differentially abundant genera in *A. ludens* when developing in *C. x aurantium* (normal) or *P. guajava* (dysbiotic); Differentially abundant genera of the normal gut microbiota of *A. ludens* compared with *A. striata* **(C)** and *A. fraterculus* **(D)**.

We detected 26 genera with differential abundance between *A. ludens* and *A. fraterculus*, and 19 differential genera between *A. ludens* and *A. striata* (Figures 3, 4A). The enriched genera in *A. ludens* were: *Pantoea*, *Gluconacetobacter*, *Ochrobactrum*, *Asaia*, *Curtobacterium*, and *Leucobacter*. Interestingly, the differential genus *Komagataeibacter* was shared between *A. striata* and *A. fraterculus*, with a higher LDA score and relative abundance in *A. striata* (Figures 3A,C, 4C,D).

### Composition of the gut microbiota of larvae of *A. ludens* developing in bitter oranges and guavas

The gut microbiota of*A. ludens* larvae exhibited a clear dysbiotic state when they fed on *P. guajava* pulp compared with *C. x aurantium*, a natural host. The PCoA showed two clusters clearly separated between both types of fruit (Figure 2B), and the PERMANOVA test detected 26% of variance explained by host type (Table 1). The LEfSe analysis detected 20 differentially abundant genera (Figure 4B). When *A. ludens* fed on guava, we observed a depletion of *Gluconobacter*, *Acetobacter*, *Weissella*, and *Dyella*. Conversely, the most incremented genera were *Pantoea* and *Enterobacter*.

**FIGURE 5:**
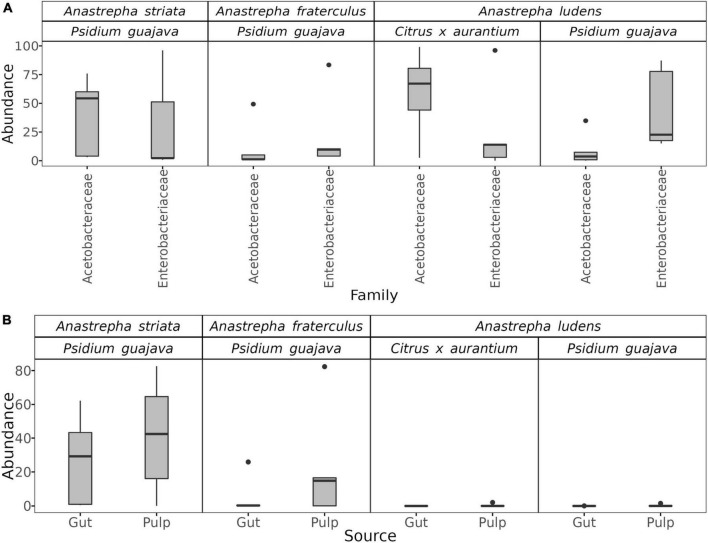
Boxplots showing the abundance distributions of the Acetobacteraceae and Enterobacteriaceae families in the gut microbiota of *Anastrepha striata*, *A*. *fraterculus* and *A*. *ludens* **(A)**, and the distribution of the genus *Komagataeibacter* in the gut and pulp microbiota according to fly species and host type **(B)**.

Surprisingly, the gut microbiota of *A. ludens* larvae reared in *C. x aurantium* was not significantly different from the *A. striata* one, and both microbiotas clustered together (Figure 2B and Table 1). LEfSe analysis detected only three differential genera between both fly species, with *Weissella* and *Gluconobacter* being overrepresented in *A. ludens*, and *Komagataeibacter* overrepresented in *A. striata* (Figure 4C). From a general taxonomic perspective, we detected a shift in the abundance ratio of Enterobacteriaceae/Acetobacteraceae families in *A. ludens* (Figure 5A), and the total absence of the genus *Komagataeibacter*, which belongs to the Acetobacteraceae family (Figure 5B).

## Discussion

Several findings stand out that we believe merit discussion: (1) Overall (considering the three fly species and two fruits studied), Alphaproteobacteria (49.8%), Gammaproteobacteria (34.1%), and Bacilli (5.1%) where the dominant groups of bacteria identified in guts of larva and fruit pulp; (2) the ubiquitous presence of *Komagataeibacter* (Acetobacteraceae), likely playing a key role in metabolizing deleterious secondary metabolites in *A*. *striata*, a tephritid fly that attacks guava when still very unripe and rife with tannins; (3) the less abundant presence of the same bacteria in *A*. *fraterculus*, another tephritid species naturally developing in guava, albeit in a much more developed ripening stage when compared to *A*. *striata*; (4) the lack of *Komagataeibacter* in the guts of *A*. *ludens* forcibly infesting ripe *P*. *guajava* fruit and the dysbiotic state of the larval microbiota of this species when developing in guavas; (5) the apparent critical role of the Enterobacteriaceae/Acetobacteraceae ratio in the fitness of the three tephritid flies studied; 6) The endosymbiont *W. pipientis wUni* was present in large numbers in *A*. *striata* and *A*. *fraterculus*, but almost inexistent in *A*. *ludens* and the pulp of guava and *C*. x *aurantium*.

At a gross taxonomic level, the gut microbiota composition was similar among the three fly species studied. Dominance of these classes, belonging to Proteobacteria and Firmicutes phyla, has also been reported in the few studies performed so far on the gut microbiota of *Anastrepha* species (Ventura et al., 2018; Gallo-Franco and Toro-Perea, 2020; Salgueiro et al., 2020; Aluja et al., 2021), and is a common pattern in the gut of phytophagous insects (Yun et al., 2014; Gales et al., 2018). The same pattern was observed in the surrounding pulp microbiota where the flies fed on. The cause of the latter could be that after egg hatch, larvae begin to move and feed in pulp, but with a limited digestive system, especially in the first instar (Carroll and Wharton, 1989). Most fruit fly larvae can only absorb liquid substrates, and thus regurgitate saliva with digestive enzymes such as proteases to dissolve solid food (Singh et al., 1988). The latter, added to the larval feces, could modify the conditions of the environment larvae face early in their development, hydrolyzing sugars, proteins, and other nutrients generating a “house foundation.” This seems to be the behavior of the three species of larvae in this study since the surrounding pulp microbiota where the larvae feed was most like the gut microbiota of each species.

We note that in this study we aimed at determining the potential role that gut microbiota plays in the survival of three species of fruit fly larvae in an environment rife with deleterious chemicals to the insects (e.g., tannins). We were interested in learning how larvae construct their niche in this “toxic” environment and transfer their gut microbiota (mostly acquired from the mother *via* vertical transmission) to their surroundings by defecating, regurgitating, and sucking. This process could ameliorate the tannin-rich pulp condition *via* the microbiota metabolism, enriching its surroundings with beneficial taxa that potentially break down toxic compounds, as we infer from our results. We fully recognize, that ideally, we should have compared the two types of pulp, totally clean, uninfested pulp, and pulp in the vicinity of the feeding larvae. However, based on literature reports (Drobya and Wisniewskib, 2018; Huang et al., 2020; Zhang et al., 2021; Malacrinò et al., 2022; Wassermann et al., 2022), we knew that pulp from an undamaged/intact (i.e., insect, pathogen, bird damage) fruit, contain a very different microbial profile (endophytic bacteria and fungi), and once a wound occurs, the microbiota profile changes rapidly. In our study we found components of the gut microbiotas of some of the fruit fly species studied in the pulp, such as the endosymbiont *Wolbachia*, which suggests that the larvae modify the pulp microbiota as *Wolbachia* is not a fruit endophyte. We nevertheless recognize that the lack of a bacterial profile of the clean guava pulp in our study could be a caveat that we will need to remediate in future studies. But importantly, the missing absolute control did not distort our results here. Furthermore, as we knew that females are very selective when choosing a particular oviposition site (i.e., fruit; Birke and Aluja, 2018), we avoided biasing our sampling approach by only working with infested fruit (i.e., circumventing the risk of sampling fruit that the females had rejected).

We were surprised/intrigued at the similarity of the gut microbiota of *A*. *striata* reared in guava and *A*. *ludens* reared in bitter orange, when this similarity would have been expected in the cases of *A*. *striata* and *A*. *fraterculus*, both attacking guava in nature (*A*. *ludens* and *A*. *striata* do not share a single host in nature). It is known that when a fruit fly larva develops in guavas, particularly in unripe fruit, it is exposed to high concentrations of secondary metabolites (Gull et al., 2012; Dos Santos et al., 2017; Monribot-Villanueva et al., 2022), especially tannins and other polyphenols, many of them toxic to the larvae (Lee et al., 2010; Birke et al., 2015; Birke and Aluja, 2018). In this sense, while *A*. *striata* prefers to oviposit into totally unripe fruit (Birke et al., 2015) that has still not reached full development (stage four or “player marble size”), *A*. *fraterculus* prefers stages five or six, green but fully developed and ripe “turning yellow” stages, respectively (Birke et al., 2015; De Oliveira et al., 2015). Thus, in fruit simultaneously infested by both species, the larvae of *A*. *fraterculus* likely ingested pulp that had been “contaminated” by the feces and saliva of larvae of *A*. *striata* as the latter started to feed much earlier in the same rearing medium (pulp).

Importantly, only three genera, *Komagataeibacter*, *Weissellla*, and *Gluconobacter*, were differential between *A. ludens* and *A. striata*. Of them, *Komagataeibacter*, an Acetic Acid Bacteria (AAB) was represented in large numbers in *A*. *striata*, in lower ones in *A*. *fraterculus* and absent in *A*. *ludens* when forcibly infesting guava and naturally infesting bitter oranges. This observation suggests a trend from up to low abundance of this bacterial genus related with the maturation stage. The ASVs of *Komagataeibacter* that we found have high sequence similarity with *Komagataeibacter intermedius* and *K. hansenii*. *K. intermedius* is used for industrial production of bacterial cellulose (Lin et al., 2016; Fernández et al., 2019), and rotten guava can be used for its enrichment and isolation (Lotfy et al., 2021). Besides, many *Komagataeibacter* species can synthesize enzymes as tannase, polyphenol oxidase, and pectinase, which are used in incomplete oxidation to metabolize a wide range of tannins and polyphenols, as gallic, tannic, gentisic, vanillic, caffeic, syringic, coumaric and ferulic acids (Usha-Rani and Anu-Appaiah, 2012; Chakravorty et al., 2016; Emiljanowicz and Malinowska-Pańczyk, 2019; Vasconcellos et al., 2019; Netzel et al., 2020). Thus, considering the robust tannin-degrading metabolic machinery among *Komagataeibacter* species (Cannazza et al., 2021), we suggest that this genus could perform important metabolic activities in *A. striata* and *A. fraterculus* that allow these flies to successfully develop in guava. We surmise that *Komagataeibacter* could degrade toxic guava compounds, allowing the development of *A. striata* and *A. fraterculus* larvae in this fruit, producing a symbiotic relationship with the benefit of adding a detoxification capacity. In addition to this presumable detoxification role, it is also possible that *Komagataeibacter* species contribute by favoring the colonization/infestation of *A. striata* and *A. fraterculus* of guavas. We hypothesize that this symbiotic relationship helps their host specificity (Myrtaceae), as guava is only infested by relatively few fruit fly species worldwide (White and Elson-Harris, 1992). In contrast, *A. ludens* establishes symbiotic relationships with a different group of AABs, and cannot acquire or coexist with *Komagataeibacter*, a fact that we now infer likely impedes its development in the tannin rich guavas.

Related to the above, we found that when *A*. *ludens* is forced to attack guavas, even fully ripe ones, the gut microbiota exhibited a clear dysbiotic state. Birke et al. (2015) and Birke and Aluja (2018) had already reported that guavas represented a strict ecological limit to the extreme polyphagy of *A*. *ludens* and that when females were forced to lay eggs into guavas, the progeny suffered severe fitness costs, such as very small pupae/adults and delayed ontogeny when compared to optimal hosts such as ones within the Rutaceae. When comparing the gut microbiota of larvae developing in guava *versus* ones developing in the natural host *C*. *x aurantium*, we observed a depletion of members within the Acetobacteraceae, specifically the genera *Gluconobacter* and *Acetobacter*, with an enrichment of representatives within the Enterobacteriaceae family, specifically the genera *Pantoea* and *Enterobacter*, resulting in a higher Enterobacteriaceae/Acetobacteraceae ratio between the dysbiotic/normal microbiota. A similar dysbiotic pattern was recently reported by Aluja et al. (2021) in the case of *A*. *ludens* attacking the marginal host *C*. *pubescens* cv. Manzano. We observed a shift in the gut microbiota, with the same increment in the Enterobacteriaceae/Acetobacteraceae ratio.

This shift could be related to the differential pupal weight of larvae developing in *C. x aurantium*, an optimal host, and *C. pubescens*, a marginal host, observed by Birke and Aluja (2018). These authors reported a very low pupal weight when *A. ludens* is forced to develop in *P. guajava* (9.5 ± 0.2 mg) when compared to the ancestral host, *C*. *edulis* (23.02 ± 0.2) and grapefruit (18.2 ± 0.2) (both Rutaceae). Based on the results obtained here on the microbiota of *A*. *ludens* larvae developing in *P*. *guajava*, as well, as those obtained by Aluja et al. (2021) in the case of *A*. *ludens* developing on the poor host *C*. *pubescens*, a plausible explanation is the depletion of AABs symbionts in these two hosts.

We note further that *C. edulis*, one of the purported ancestral hosts of *A*. *ludens*, can be considered a “sugar bomb” with high carbohydrate content and low protein levels [carbohydrate/protein ratio of 18.4 (16.6/0.9%)], contrasting to what happens in *P. guajava* [8.7 (15.8/1.8%)] and *C. pubescens* [6.3 (6.32/1%) (Morton and Dowling, 1987; Rivera et al., 2010; Birke and Aluja, 2018)]. That is, the latter fruit species have more than two times the amount of protein content than *C*. *edulis*, which is a common nutrient trend in native/domesticated fruit (Aluja and Mangan, 2008). Phytophagous specialists mostly prefer hosts that maximize offspring fitness; specificity also guarantees the same nutritional availability generation after generation, producing a “phylogenetic-specific” phytophagia, since phylogenetically related plant species have similar nutritional compounds and similar types of secondary metabolites and volatiles (Birke and Aluja, 2018). On the other hand, the ability to grow adequately in nitrogen poor sources (high C:N ratio) could be key for the polyphagia exhibited by *A*. *ludens*, reducing the need for specificity as any host with high carbohydrate content will be adequate, granted there are no toxic secondary metabolites. Thus, we hypothesize that *A. ludens* lacks a phylogenetic-specific host-fly relationship but could rather exhibit a nutritional-specific host-fly relationship, capable of infesting any host with low toxicity and a high C:N ratio in nutrient composition. The type of bacterial relationships found in the case of *A*. *ludens* and *C*. x *aurantium* here, plus the ones recently reported by Aluja et al. (2021) in the case of other hosts, lends support to our hypothesis.

The apparent critical role of the Enterobacteriaceae/Acetobacteraceae ratio in the fitness in the of the three tephritid flies studied represents an interesting phenomenon. Many insects that develop in hosts with high C:N ratios overcome the nitrogen deficiency *via* their gut microbiota (Bar-Shmuel et al., 2020; Ren et al., 2022). The gut microbiota can make the nitrogen available to the insect host *via* biological nitrogen fixation (BNF), where nitrogen-fixing bacteria, using the nitrogenase complex can transform the atmospheric dinitrogen into ammonia, which can be later assimilated as the non-essential amino acids glutamine and glutamate (Kneip et al., 2007; Ren et al., 2022). This type of symbiosis has been reported in the tephritid flies *Ceratitis capitata* and *Bactrocera tryoni*, involving bacteria of the genera *Enterobacter* and *Klebsiella* (Murphy et al., 1994; Behar et al., 2005). Here, as noted before, we found high abundance of Acetic Acid Bacteria (AAB) in the three *Anastrepha* species. AAB’s integrate the Acetobacteraceae family, and the genera *Acetobacter*, *Gluconacetobacter*, *Gluconobacter*, *Asaia*, and *Saccharibacter* are known to establish symbiotic interactions with insects within Diptera, Hemiptera, and Hymenoptera developing in sugar rich diets (Crotti et al., 2009, 2010; Bar-Shmuel et al., 2020). To date, among all Acetobacteraceae several representatives of the genera *Gluconacetobacter, Acetobacter, Komagataeibacter, Swaminathania, Asaia*, and *Acetobacter* have been reported as nitrogen fixing bacteria (Cavalcante and Dobereiner, 1988; Pedraza, 2008; Reis and dos Teixeira, 2015; Dwivedi, 2020).

We also observed an interesting shift in the gut microbiota of *A. ludens*, with the genera *Gluconobacter* and *Acetobacter* being enriched when larvae developed in *C.* x *aurantium* but notably depleted when forcibly infesting *P. guajava*. This observation is congruent with our recently published work (Aluja et al., 2021), where we show that the infestation of *C*. *pubescens*, a marginal/poor host only used in extreme drought conditions, produces a high fitness cost resulting in a depletion of Acetobacteraceae and a concomitant enrichment of Enterobacteriaceae (Aluja et al., 2021). A similar observation has been reported in the mosquitoes *Anopheles stephensi* and *A. gambiae*, where the loss of *Asaia spp*. (an AAB) delays the development of its larvae (Chouaia et al., 2012; Mitraka et al., 2013). The most enriched Enterobacteriaceae when *A. ludens* forcibly infested *P. guajava* was *Pantoea*. This genus comprises a versatile group of species that are common commensals or symbionts of plants and insects but that can also become parasitic or play key functions in habitat restoration and pesticide degradation (Walterson and Stavrinides, 2015; Dutkiewicz et al., 2016a,b). Given the versatile ecology of *Pantoea*, we could envisage it as a “Jekyll and Hyde” ectosymbiont, that under an *A. ludens* physiological imbalance condition, starts to proliferate and ultimately develops the dysbiotic condition we detected. In contrast to AAB, *Pantoea* comprise anaerobic or facultative anaerobic bacteria, thus, a shift between AAB/*Pantoea* could indicate a shift in the redox condition or oxygen availability for *A. ludens*, but its mechanism of action did not become obvious or could not be formally disentangled in this study.

Finally, we found a high coverage of ASVs belonging to *Wolbachia pipientis* in the guts of *A. striata* and *A. fraterculus*, but notably the presence of this endosymbiont was almost inexistent in the pulp in which both flies feed on. *Wolbachia* is a common endosymbiont of insects and its vertical transmission in both fly species has been documented (Martínez et al., 2012; Conte et al., 2019; Mateos et al., 2020); thus, the presence of *Wolbachia* in pulp likely originated from the guts of the larvae when defecating into the pulp. We also detected *Wolbachia* with very low coverage in the guts of *A. ludens* and in the pulp of *C. x aurantium*, but importantly, this endosymbiont was not found in the pulp of *P. guajava* when *A*. *ludens* forcibly infested this fruit. The origin of *Wolbachia* in *A. ludens* is not clear, but according to a previous report (Martínez et al., 2012), this endosymbiont seems unable to infect or prevail in this fly, but additional studies are necessary in this respect.

In conclusion, changes in microbiota composition of the gut of *A. striata*, *A. fraterculus* and *A. ludens* are related to its phytophagous habits and host use capabilities. Beneficial traits from the microbiota are different in *A. striata* and *A. fraterculus* compared with *A. ludens*. In the first two species, the specificity and capability to use a host rich in tannins is likely supported by AABs within *Komagateibacter*. We thus surmise that this relationship promotes the host specificity observed in nature (Birke et al., 2015), which secures the same nutritional availability in each generation, and excludes competitors that cannot infest *P. guajava* rife with tannins and other deleterious polyphenols. On the other hand, we now know that the fact that *A. ludens* cannot effectively develop in guava could be likely related to the adverse effects of toxic metabolites in this fruit that this polyphagous insect cannot metabolize, either because the metabolic machinery is missing, or the appropriate gut microbiota is not present. In this sense, our prediction that the deleterious guava pulp would generate a dysbiotic gut microbiota condition, was confirmed. We suggest that *A. ludens* harbors a microbiota which mainly ameliorates a poor nitrogen content in its hosts. Such microbiota allows this species to infest many hosts with a deficient nutritional content, particularly low nitrogen content, thus promoting its polyphagia. Further functional studies for the detection of nitrogenase activity are still needed, as well as tests with gnotobiotic flies to determine the specific functions provided by the gut microbiota of each fly species. These, as well as many other outstanding questions related with the role microorganisms possibly play in metabolizing toxic chemicals in plants, offer fertile ground for future research (Hansen and Moran, 2014).

## Data availability statement

The raw data presented in this study are available in the NCBI repository (BioProject PRJNA684483). Processed data to replicate the analysis are in the Supplementary material.

## Author contributions

MA, AM, and MO-S: conceptualization. MO-S and DC-G: data curation and formal analysis. MA: funding acquisition. MO-S, AA-M, DC-G, and MA: investigation and methodology. MA and AA-M: project administration and resources. DC-G: software and visualization. MA, AA-M, EI-L, DD, and AM: supervision. MO-S, MA, and DC-G: writing—original draft. MA, DC-G, MO-S, AM, AA-M, DD, and EI-L: writing—review and editing. All authors contributed to the article and approved the submitted version.

## Footnotes

1
https://emea.illumina.com/content/dam/illumina-support/documents/documentation/chemistry_documentation/16s/16s-metagenomic-library-prep-guide-15044223-b.pdf
