# Supplementary material for: Bitter friends are not always toxic: The loss of acetic acid bacteria and the absence of Komagataeibacter in the gut microbiota of the polyphagous fly Anastrepha ludens could inhibit its development in Psidium guajava in contrast to A. striata and A. fraterculus that flourish in this host
Source: Front Microbiol. 2022 Sep 28;13:979817. doi: 10.3389/fmicb.2022.979817 (PMC9554433; doi:10.3389/fmicb.2022.979817)
Supplement: Supplementary file 1 [file Data_Sheet_1.ZIP › R_code.html]

Bitter friends are not always toxic: the loss of acetic acid bacteria and the absence of Komagataeibacter in the gut microbiota of the polyphagous fly Anastrepha ludens could inhibit its development in Psidium guajava in contrast to A. striata and A. fraterculus that flourish in this host


# Bitter friends are not always toxic: the loss of acetic acid bacteria and the absence of *Komagataeibacter* in the gut microbiota of the polyphagous fly *Anastrepha ludens* could inhibit its development in *Psidium guajava* in contrast to *A. striata* and *A. fraterculus* that flourish in this host

#### Crated by Daniel Cerqueda-García

## Procesing reads in QIIME2

```
### Import data

qiime tools import --type 'SampleData[PairedEndSequencesWithQuality]' --input-path raw_fastq_files --input-format CasavaOneEightSingleLanePerSampleDirFmt --output-path paired_end.qza

### Resolve ASVs with DADA2

qiime dada2 denoise-paired --i-demultiplexed-seqs paired_end.qza --p-trim-left-f 20 --p-trim-left-r 20 --p-trunc-len-f 270 --p-trunc-len-r 200 --p-n-threads 40 --output-dir dada_out --verbose &

### Taxonomic classification
### Download SILVA 132 database from the QIIME2 data resource (https://docs.qiime2.org/<version>/data-resources/) 

cd dada_out

qiime feature-classifier classify-consensus-vsearch --i-query  representative_sequences.qza --i-reference-reads SILVA132_16sOnly.qza --i-reference-taxonomy taxonomy132_16sOnly_7l.qza --p-threads 40 --output-dir vsearch_taxonomy --verbose 

### Build phylogeny

qiime phylogeny align-to-tree-mafft-fasttree --i-sequences representative_sequences.qza --p-n-threads 40 --output-dir phylogeny

### Export results


qiime tools export --input-path table.qza --output-path exported-feature-table

qiime tools export --input-path vsearchtaxonomy/classification.qza --output-path taxonomy
qiime tools export --input-path phylogeny/rooted_tree.qza --output-path phylogeny
```

## Load and process data in R

```
###Load required packages

library(phyloseq)
library(ggplot2)
library(vegan)
library(tidyverse)
library(metagMisc)

### Load biom file, taxonomy and rooted_tree

data <- import_biom("new_otu_table.biom","tree.nwk")
sampledata <- import_qiime_sample_data("metadata.txt")
data <- merge_phyloseq(data,sample_data)

### Filter out ASVs of Mitochondria and Chloroplast

proks<-which(tax_table(data) == "Mitochondria" | tax_table(data) == "Chloroplast" , arr.ind=T)%>% rownames()
data<-prune_taxa(taxa_names(data)[!taxa_names(data) %in% proks],data)

### Separate data set with Wolbachia

wolbachia_data <- subset_taxa(data, Genus == "Wolbachia")

### Separate data set with gut microbiota

microbiota_data <- subset_taxa(data, Genus != "Wolbachia")

### Normalize data sets with CSS 

wolbachia_data_css <- phyloseq_transform_css(wolbachia_data, norm = TRUE, log = FALSE)

microbiota_data_css <- phyloseq_transform_css(microbiota_data, norm = TRUE, log = FALSE)

### PCoA with UniFrac distances metrics

pcoa_microbiota <- ordination(microbiota_data_css, "PCoA","uunifrac")

### Calculate UniFrac distance metrics

uunifrac_microbiota <- ordinate(microbiota_data_css,"uunifrac")

wunifrac_microbiota <- ordinate(microbiota_data_css, "wunifrac")

### Calculate PERMANOVA in one way mode: flies in P. guajaba.

subset_samples(microbiota_data_css, Plant_host=="Psidium_guajava" & Isolation_source == "Gut") %>% filter_taxa(function(x) sum(x) > 0,T) %>% adonis(uunifrac_microbiota ~ Host, as.data.frame(sample_data(.)))

subset_samples(microbiota_data_css, Plant_host=="Psidium_guajava" & Isolation_source == "Gut")  %>% filter_taxa(function(x) sum(x) > 0, T) %>% adonis(wunifrac_microbiota ~ Host, as.data.frame(sample_data(.)))


### Create a function to apply adonis in pairwise mode

pairwise.adonis.physeq <- function(x,factors,dist.m="wunifrac", perm=999,p.adjust.m ='BH'){
co <- combn(unique(as.character(sample_data(x)[,factors][[1]])),2)
pairs <- c()
F.Model <- c()
R2 <- c()
p.value <- c()
for (elem in 1:ncol(co)){ 
cosa<-c(co[1,elem],co[2,elem])
filtered<-prune_samples(sample_names(x)[which( sample_data(x)[,factors][[1]] %in% cosa)],x)
filtered<- filter_taxa(filtered, function(x) sum(x) > 0, TRUE)
x1=distance(filtered,dist.m)
form<-paste("x1","~",factors)
ad = adonis(as.formula(form),as(sample_data(filtered),"data.frame"),perm=perm);
#print(cosa)
pairs = c(pairs,paste(co[1,elem],'vs',co[2,elem]));
F.Model =c(F.Model,ad$aov.tab[1,4]);
R2 = c(R2,ad$aov.tab[1,5]);
p.value = c(p.value,ad$aov.tab[1,6])
}
p.adjusted = p.adjust(p.value,method=p.adjust.m)
sig = c(rep('',length(p.adjusted)))
sig[p.adjusted <= 0.05] <-'.'
sig[p.adjusted <= 0.01] <-'*'
sig[p.adjusted <= 0.001] <-'**'
sig[p.adjusted <= 0.0001] <-'***'
pairw.res = data.frame(pairs,F.Model,R2,p.value,p.adjusted,sig)
print("Signif. codes:  0 ‘***’ 0.001 ‘**’ 0.01 ‘*’ 0.05 ‘.’ 0.1 ‘ ’ 1")
return(pairw.res)
}

### Calculate PERMANOVA in pairwise mode: flies in P. guajaba.


subset_samples(microbiota_data_css, Plant_host=="Psidium_guajava" & Isolation_source == "Gut")  %>% filter_taxa(function(x) sum(x) > 0, T) %>% pairwise.adonis.physeq(.,factors = "Host", dist.m = "wunifrac")

subset_samples(microbiota_data_css, Plant_host=="Psidium_guajava" & Isolation_source == "Gut")  %>% filter_taxa(function(x) sum(x) > 0, T) %>% pairwise.adonis.physeq(.,factors = "Host", dist.m = "uunifrac")


### Calculate PERMANOVA in pairwise mode: A. ludens in C auriantum, A. striata and A. fraterculus in P. guajaba.

ludens_in_guava_ids <- subset_samples(datos_sn_wolbachia_CSS, Isolation_source == "Gut") %>%   subset_samples(Host=="Anastrepha_ludens" & plant_host == "Psidium_guajava") %>% sample_names 

subset_samples(datos_sn_wolbachia_CSS, Isolation_source == "Gut") %>% subset_samples(!X.SampleID %in% ludens_in_guava_ids) %>% filter_taxa(function(x) sum(x) > 0, T) %>% pairwise.adonis.physeq(.,factors = "Host",dist.m = "uunifrac")

subset_samples(datos_sn_wolbachia_CSS, Isolation_source == "Gut") %>% subset_samples(!X.SampleID %in% ludens_in_guava_ids) %>% filter_taxa(function(x) sum(x) > 0, T) %>% pairwise.adonis.physeq(., factors = "Host",dist.m = "wunifrac")

### Calculate PERMANOVA gut vs pulp for each species.
#A. fraterculus
subset_samples(microbiota_data_css, Host=="Anastrepha_fraterculus")  %>% filter_taxa(function(x) sum(x) > 0, T) %>% pairwise.adonis.physeq(., factors = "Isolation_source",dist.m = "uunifrac") 

subset_samples(microbiota_data_css, Host=="Anastrepha_fraterculus")  %>% filter_taxa(function(x) sum(x) > 0, T) %>% pairwise.adonis.physeq(., factors = "Isolation_source",dist.m = "wunifrac")

#A. striata
subset_samples(microbiota_data_css, Host=="Anastrepha_striata")  %>% filter_taxa(function(x) sum(x) > 0, T) %>% pairwise.adonis.physeq(., factors = "Isolation_source",dist.m = "uunifrac") 

subset_samples(microbiota_data_css, Host=="Anastrepha_striata")  %>% filter_taxa(function(x) sum(x) > 0, T) %>% pairwise.adonis.physeq(., factors = "Isolation_source",dist.m = "wunifrac")

#A. ludens in P. guajaba
subset_samples(microbiota_data_css, Host=="Anastrepha_ludens" & Plant_host == "Psidium_guajava")  %>% filter_taxa(function(x) sum(x) > 0, T) %>% pairwise.adonis.physeq(., factors = "Isolation_source",dist.m = "wunifrac")

subset_samples(microbiota_data_css, Host=="Anastrepha_ludens" & Plant_host == "Psidium_guajava")  %>% filter_taxa(function(x) sum(x) > 0, T) %>% pairwise.adonis.physeq(., factors = "Isolation_source",dist.m = "uunifrac")

#A. ludens in C. aurantium

subset_samples(microbiota_data_css, Host=="Anastrepha_ludens" & Plant_host == "Citrus_x_aurantium")  %>% filter_taxa(function(x) sum(x) > 0, T) %>% pairwise.adonis.physeq(., factors = "Isolation_source",dist.m = "wunifrac")

subset_samples(microbiota_data_css, Host=="Anastrepha_ludens" & Plant_host == "Citrus_x_aurantium")  %>% filter_taxa(function(x) sum(x) > 0, T) %>% pairwise.adonis.physeq(., factors = "Isolation_source",dist.m = "uunifrac")


### Export data to LEfSe

#Create function to export paired data using phyloseqCompanion
library(phyloseqCompanion)

genus_lefse_pairs <- function(x, FILE, factors, LEV="Genus"){
co <- combn(unique(as.character(sample_data(x)[,factors][[1]])),2)
for(elem in 1:ncol(co)){ 
cosa<-c(co[1,elem],co[2,elem])
filtered<-prune_samples(sample_names(x)[which( sample_data(x)[,factors][[1]] %in% cosa)],x)
filtered<- filter_taxa(filtered, function(x) sum(x) > 0, TRUE)
filtered <- tax_glom(filtered,LEV)
tax_table(filtered) <- tax_table(filtered)[,LEV]
phyloseq2lefse(filtered,factors,paste0(FILE,"_",co[1,elem],"_",co[2,elem],".txt"),taxa.levels=LEV)
print(paste0(FILE,"_",co[1,elem],"_",co[2,elem],".txt file created"))
}
}


#Export paired data of the gut of the three flies in P. guajava

subset_samples(microbiota_data_css, Plant_host=="Psidium_guajava" & Isolation_source == "Gut")  %>% filter_taxa(function(x) sum(x) > 0, T) %>% genus_lefse_pairs(.,x = "GUT_guava",factors = "HOST")


#Export gut data of A. ludens in P. guajaba vs C. aunrantium

subset_samples(microbiota_data_css, Host=="Anatrepha_ludens" & Isolation_source == "Gut")  %>% filter_taxa(function(x) sum(x) > 0, T) %>% phyloseq2lefse(., "Plant_host", "ludens_to_lefse.txt", "Genus")

### Output data now can be loaded to galaxy for the LEfSe analysis.
```
