# Supplementary material for: Bitter friends are not always toxic: The loss of acetic acid bacteria and the absence of Komagataeibacter in the gut microbiota of the polyphagous fly Anastrepha ludens could inhibit its development in Psidium guajava in contrast to A. striata and A. fraterculus that flourish in this host
Source: Front Microbiol. 2022 Sep 28;13:979817. doi: 10.3389/fmicb.2022.979817 (PMC9554433; doi:10.3389/fmicb.2022.979817)
Supplement: Supplementary file 2 [file Data_Sheet_2.docx]

Supplementary Material

## Supplementary Figures


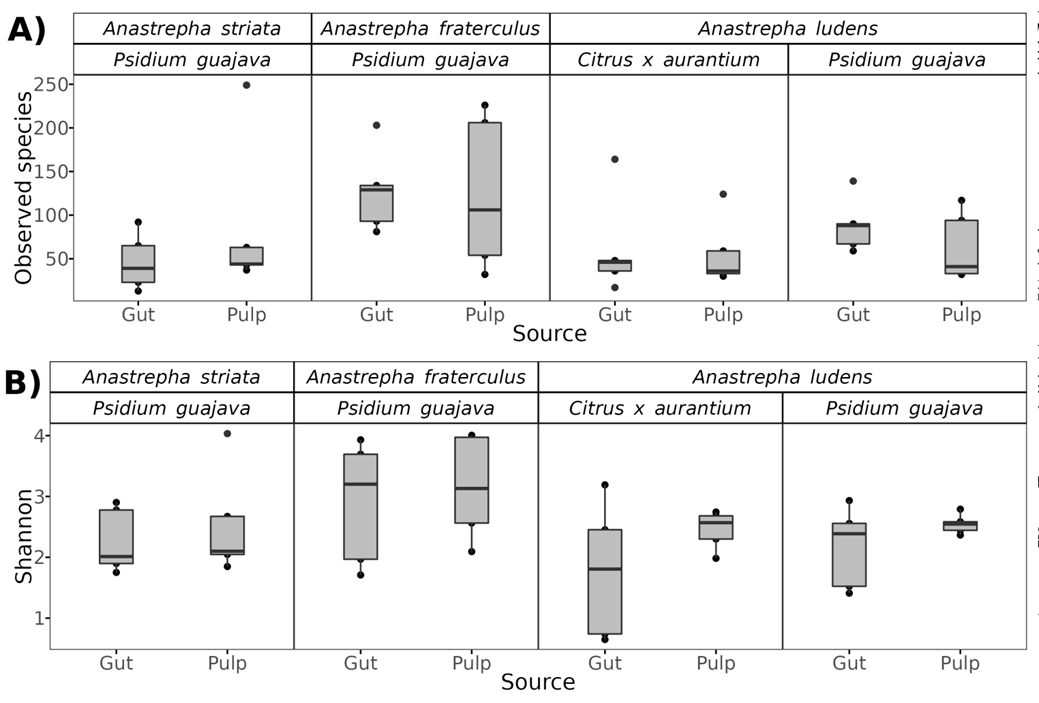


**Supplementary Figure 1.** Alpha diversity indexes: observed species (A) and Shannon (B) calculated in the gut microbiota of *Anastrepha* *striata*, *A*. *fraterculus* and *A*. *ludens* and the pulp where larvae fed on.

Supplementary Tables

Table S1. PERMANOVA results of the gut microbiota of *Anastrepha ludens* (in *C. x aurantium*), *A. striata* (in *P. guajava*) and *A. fraterculus* (in *P. guajava*) calculated with the UniFrac distance matrix.

| **Metric** | **F. Model** | **R^2^** | **p-value** |
| --- | --- | --- | --- |
| Weighted | 2.22 | 0.27 | 0.046 |
| Unweighted | 2.81 | 0.31 | 0.002 |

Table S2. Pairwise PERMANOVA comparisons of the gut microbiota of *Anastrepha ludens, A. striata*, and *A. fraterculus* larvae developing in *P. guajava* or *C. x auriantum.* P-values were adjusted with the BH method.

| **Gut microbiota in *P. guajava*** | | | |
| --- | --- | --- | --- |
| **Comparison** | **F. model** | **R^2^** | **adj. p-value** |
| *A. striata / A. fraterculus* | 2.74 | 0.25 | 0.04 |
| *A. striata / A. ludens* | 2.43 | 0.23 | 0.02 |
| *A. fraterculus / A. ludens* | 3.26 | 0.28 | 0.02 |
| **Gut microbiota of *A. ludens* in *C. x auriantum*** | | | |
| **Comparison** | **F. model** | **R^2^** | **adj. p-value** |
| *A. ludens* / *A. striata vs* | 1.09 | 0.12 | 1 |
| *A. ludens* / *A. fraterculus* | 3.28 | 0.29 | 0.04 |
| **Gut microbiota of flies compared with the pulp where each fly fed on** | | | |
| ***Comparison*** | **F. model** | **R2** | **adj. p-value** |
| *A. ludens /* Pulp *P. guajava* | 2.09 | 0.20 | 0.07 |
| *A. ludens /* Pulp *C. x aurantium* | 1.19 | 0.12 | 0.29 |
| *A. striata/* Pulp *P. guajava* | 1.43 | 0.15 | 0.15 |
| *A. fraterculus/* Pulp *P. guajava* | 1.46 | 0.15 | 0.13 |

Table S3. Statistics of number or reads for each data set analyzed in this study.

| **Fly species** | **Plant host** | **Isolation source** | **min.** | **max.** | **mean** | **Type of data** |
| --- | --- | --- | --- | --- | --- | --- |
| *Anastrepha_fraterculus* | *Psidium_guajava* | Gut | 78967 | 139437 | 118690.4 | Complete set |
| *Anastrepha_fraterculus* | *Psidium_guajava* | Pulp | 67901 | 166173 | 124615.8 | Complete set |
| *Anastrepha_ludens* | *Citrus_x_aurantium* | Gut | 81745 | 160857 | 111494.2 | Complete set |
| *Anastrepha_ludens* | *Citrus_x_aurantium* | Pulp | 75473 | 167954 | 108718.4 | Complete set |
| *Anastrepha_ludens* | *Psidium_guajava* | Gut | 85690 | 139673 | 115004.2 | Complete set |
| *Anastrepha_ludens* | *Psidium_guajava* | Pulp | 113479 | 160008 | 144207.2 | Complete set |
| *Anastrepha_striata* | *Psidium_guajava* | Gut | 72622 | 168179 | 120406.8 | Complete set |
| *Anastrepha_striata* | *Psidium_guajava* | Pulp | 157502 | 254678 | 222062 | Complete set |
| *Anastrepha_fraterculus* | *Psidium_guajava* | Gut | 78626 | 139393 | 114842.4 | Clean set |
| *Anastrepha_fraterculus* | *Psidium_guajava* | Pulp | 57143 | 156491 | 103346.2 | Clean set |
| *Anastrepha_ludens* | *Citrus_x_aurantium* | Gut | 74539 | 160763 | 96864 | Clean set |
| *Anastrepha_ludens* | *Citrus_x_aurantium* | Pulp | 27825 | 150299 | 57598.6 | Clean set |
| *Anastrepha_ludens* | *Psidium_guajava* | Gut | 85668 | 139313 | 114339 | Clean set |
| *Anastrepha_ludens* | *Psidium_guajava* | Pulp | 12360 | 152454 | 115655.4 | Clean set |
| *Anastrepha_striata* | *Psidium_guajava* | Gut | 70188 | 166536 | 97366.8 | Clean set |
| *Anastrepha_striata* | *Psidium_guajava* | Pulp | 35726 | 199215 | 118293.4 | Clean set |
| *Anastrepha_fraterculus* | *Psidium_guajava* | Gut | 21166 | 75582 | 46295.6 | Microbiota set |
| *Anastrepha_fraterculus* | *Psidium_guajava* | Pulp | 57007 | 156438 | 103266 | Microbiota set |
| *Anastrepha_ludens* | *Citrus_x_aurantium* | Gut | 74539 | 160658 | 95486.2 | Microbiota set |
| *Anastrepha_ludens* | *Citrus_x_aurantium* | Pulp | 27796 | 150299 | 57589 | Microbiota set |
| *Anastrepha_ludens* | *Psidium_guajava* | Gut | 85545 | 137353 | 113219.6 | Microbiota set |
| *Anastrepha_ludens* | *Psidium_guajava* | Pulp | 12360 | 152454 | 115655.4 | Microbiota set |
| *Anastrepha_striata* | *Psidium_guajava* | Gut | 593 | 59420 | 29835.2 | Microbiota set |
| *Anastrepha_striata* | *Psidium_guajava* | Pulp | 35711 | 199204 | 118257.6 | Microbiota set |
| *Anastrepha_fraterculus* | *Psidium_guajava* | Gut | 28845 | 118227 | 68546.8 | Endosymbiont set |
| *Anastrepha_fraterculus* | *Psidium_guajava* | Pulp | 53 | 136 | 80.2 | V |
| *Anastrepha_ludens* | *Citrus_x_aurantium* | Gut | 0 | 6550 | 1377.8 | Endosymbiont set |
| *Anastrepha_ludens* | *Citrus_x_aurantium* | Pulp | 0 | 29 | 9.6 | Endosymbiont set |
| *Anastrepha_ludens* | *Psidium_guajava* | Gut | 33 | 3347 | 1119.4 | Endosymbiont set |
| *Anastrepha_ludens* | *Psidium_guajava* | Pulp | 0 | 0 | 0 | Endosymbiont set |
| *Anastrepha_striata* | *Psidium_guajava* | Gut | 10768 | 161828 | 67531.6 | Endosymbiont set |
| *Anastrepha_striata* | *Psidium_guajava* | Pulp | 0 | 98 | 35.8 | Endosymbiont set |

**Supplementary methods**

We used PICRUSt2 (Douglas et al., 2020) to infer the metabolic pathways of gut microbiota. The normalized abundance table of ASVs was used as input to the PICRUSt2 pipeline using the default parameters (full pipeline <https://github.com/picrust/picrust2/wiki/Full-pipeline-script>). In the resulting pathway abundance table, we searched for the metabolism involved in tannin/other polyphenols and nitrogenase degradation. The pathway abundance between fly species and host plant was tested with a U-Mann Whitney test.


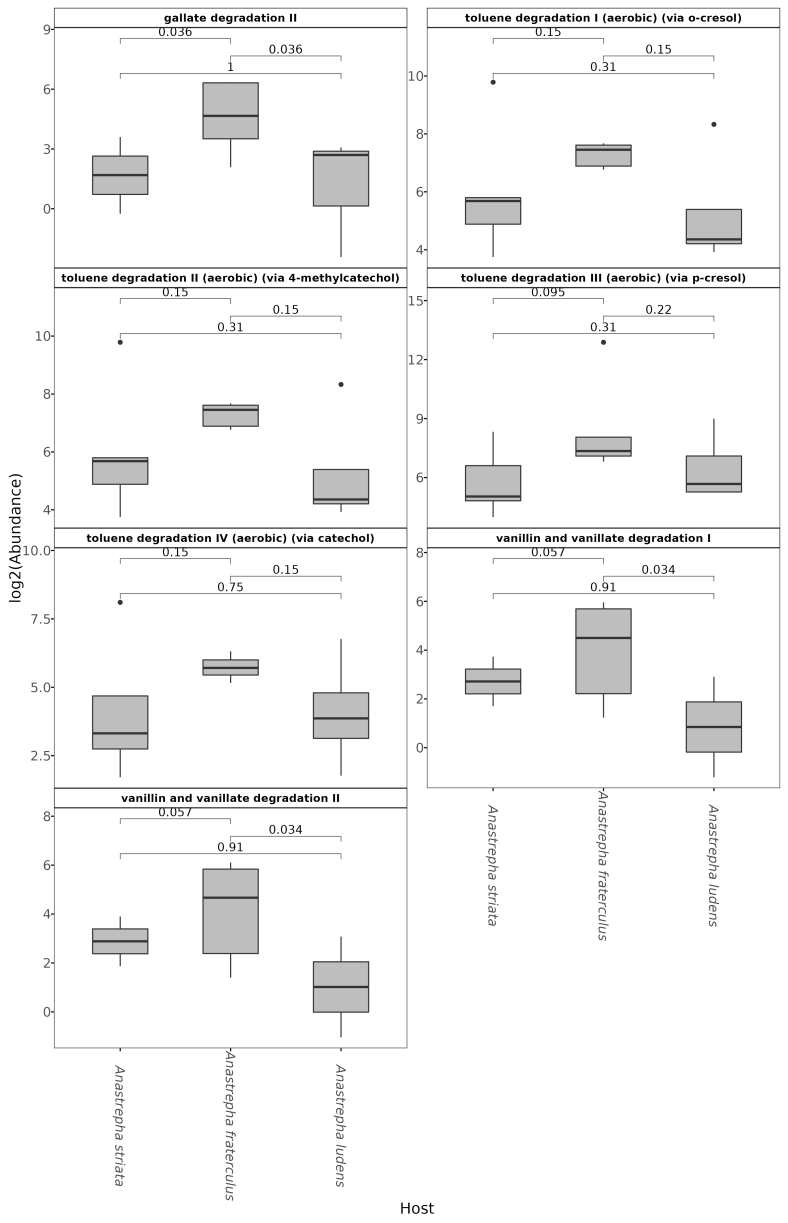
**Supplementary results**

**Figure S2.** Boxplots of the inferred metabolic pathways involved in tannin and polyphenol degradation in the three flies, *A*. *ludens* in *C*. *aurantium*, *A*. *striata*, and *A*. *fraterculus* in *P*. *guajava*. P-values of the U-Mann Whitney tests in pairwise comparisons are shown. Log2(Abundance) corresponds to log 2 of the pathway abundance per read.


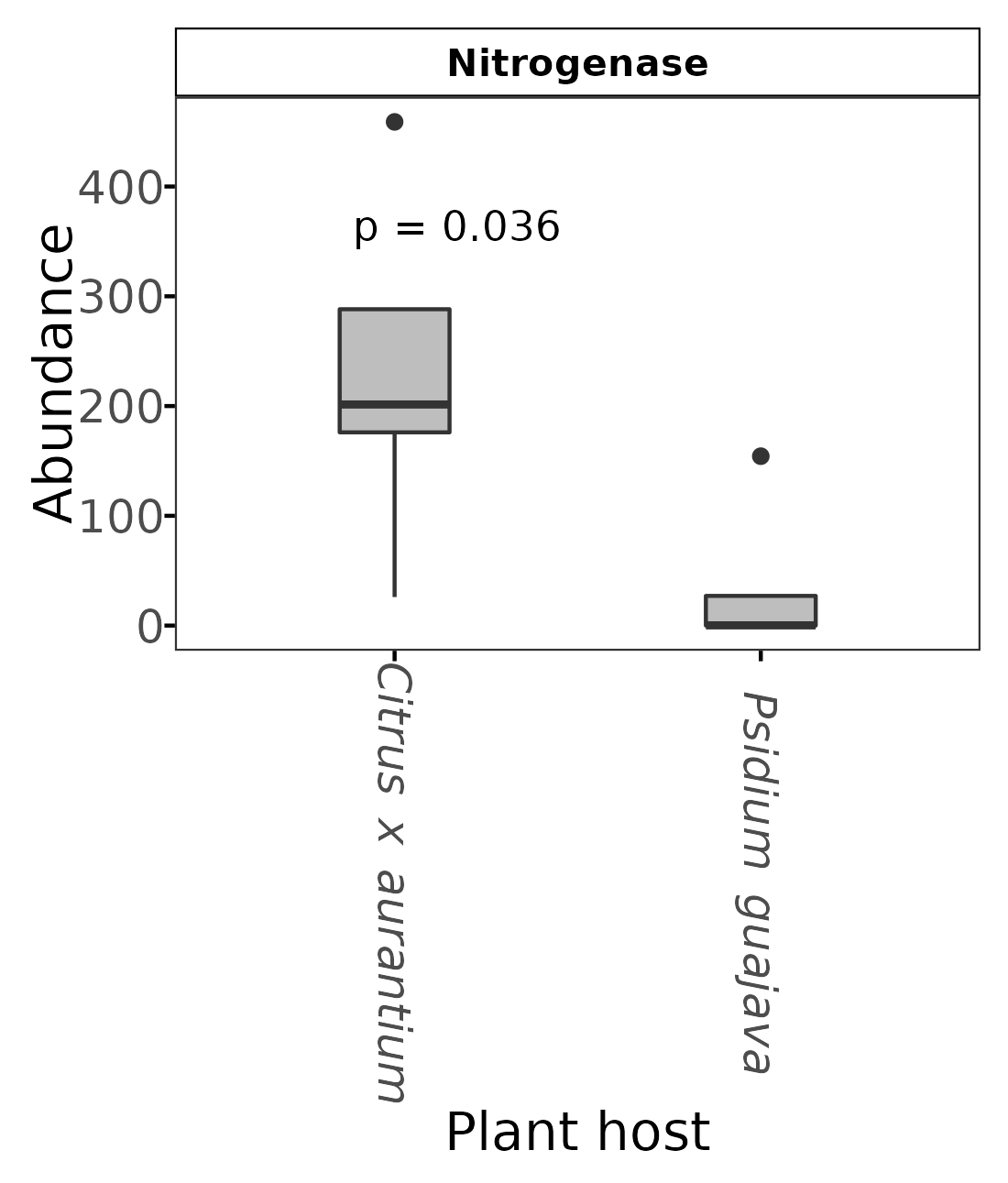


**Figure S3**. Boxplot of the abundance of nitrogenase inferred with PICRUSt2 of *A*. *ludens* fed on *C*. *aurantium* or *P*. *guajava*. P-values correspond to the U-Mann Whitney tests. Abundance corresponds to enzyme abundance per read.

**Supplementary references**

Douglas, G. M., Maffei, V. J., Zaneveld, J. R., Yurgel, S. N., Brown, J. R., Taylor, C. M., ... & Langille, M. G. (2020). PICRUSt2 for prediction of metagenome functions. *Nature biotechnology*, *38*(6), 685-688.
